# Effects of different ablation strategies on long-term left atrial function in patients with paroxysmal atrial fibrillation: a single-blind randomized controlled trial

**DOI:** 10.1038/s41598-019-44168-5

**Published:** 2019-05-22

**Authors:** Ling You, Lixia Yao, Bolun Zhou, Lili Jin, Honglin Yin, Jinglan Wu, Guangli Yin, Ying Yang, Chenfeng Zhang, Yue Liu, Ruiqin Xie

**Affiliations:** 1 Division of Cardiology, The Second Hospital of Hebei Medical University, Shijiazhuang, Hebei People’s Republic of China; 2 Xiangya School of Medicine, Central South University, Changsha, People’s Republic of China

**Keywords:** Interventional cardiology, Outcomes research

## Abstract

Restoration of sinus rhythm in atrial fibrillation (AF) by radiofrequency catheter ablation (RFCA) is associated with a transient stunning of left atrial (LA) function. However, the long-term effects of different ablation strategies on LA function remain undetermined. We performed randomized controlled trial to evaluate the effects of RFCA, cryoablation, and 3D mapping-guided cryoablation on LA function of proximal AF patients within 1 year. The 3D mapping-guided cryoablation was defined as a maximum of two cryoablation procedures for each pulmonary vein accompanied by RFCA for additional points until complete pulmonary vein isolation was achieved. Conventional and speckle tracking echocardiographic analyses were performed to evaluate LA function. Among the 210 patients (70 in each group) included, a trend of decreasing LA systolic and diastolic function was observed in all groups, as evidenced by decreases in peak A-wave velocity, the global LA peak systolic strain, the peak strain rate, the peak early diastolic strain rate, and the peak late diastolic strain rate within 7 days to 3 months after ablation followed by gradual recovery thereafter. However, the temporal changes in the above four strain parameters among the three groups did not differ significantly within 1 year after ablation (all p > 0.05). Parameters of the LA emptying fraction and LA dimensions were not significantly affected. These results suggested that stunning of LA function occurred within 7 days to 3 months after ablation, and different strategies of AF ablation did not differentially affect the temporal changes in LA function up to 1 year after ablation.

## Introduction

Atrial fibrillation (AF) is a common atrial arrhythmia that is prevalent particularly in the elderly^1^. Patients with AF have a significantly increased risk of thromboembolism events and heart failure, which makes the disease an important cause of mortality worldwide^2^. Currently, pulmonary vein isolation (PVI) by means of catheter ablation has become an important treatment for patients with drug-refractory paroxysmal AF (PAF)^3^. Conventionally, PVI is achieved by radiofrequency catheter ablation (RFCA). It has been recognized that restoration of sinus rhythm in AF is associated with a transient stunning of left atrial (LA) function, which may be associated with an increased risk of thromboembolic events^4,5^. Our recent study in PAF patients undergoing RFCA indicated that dramatically reduced LA function occurred within 7 days after ablation and was accompanied by an increased prothrombotic tendency^6^. However, the long-term changes in LA function following RFCA remain unclear.

Cryoballoon ablation has emerged as a novel ablation strategy in patients with PAF^7^. By single energy release for lesions encircling the pulmonary vein (PV)^8^, the efficacy of cryoballoon ablation for AF has been confirmed in previous studies^9^. However, for some AF patients (15%^10^ and 28%^11^ according to previous studies), unrecognized incomplete PVI may persist despite the application of a standardized protocol for cryoablation and the use of a circular mapping catheter to record the LA-PV potential connections. For these patients, 3D mapping-guided cryoablation complemented with RFCA of additional points may improve the efficacy of cryoablation. However, the long-term temporal changes in LA function of AF patients following cryoablation and cryoablation complemented with RFCA, to the best of our knowledge, have been rarely evaluated. The aim of the study was to evaluate the effects of cryoablation, 3D mapping-guided cryoablation, and RFCA on long-term atrial function in patients with PAF.

## Results

### Baseline characteristics

Overall, 210 patients (mean age: 59.1 years) with PAF were included in the current study, with 70 randomized to each group. The baseline characteristics of the included patients are shown in Table 1. All of the included patients achieved complete PVI during the ablation procedures and were followed up for 12 months except for one patient in the RF group. This patient withdrew from the study 3 months after the procedure because of gastrointestinal bleeding that was not related to the PVI treatment. In the Freeze group, the first generation cryoballoon was applied in 29 patients (41.4%), whereas in the Freeze 3D group, the first generation cryoballoon was applied in eight patients (11.4%). All other patients in these two groups received ablation using the second generation cryoballoon. Eighteen patients in the Freeze 3D group did not achieve complete PVI, despite application of the cryoablation twice for each PV. Accordingly, RFCA was applied for the additional points until complete PVI was achieved. Some perioperative outcomes for patients of the three groups are also presented in Table 1. The treatment in the Freeze group was associated with significantly higher doses of surgical X-ray during ablation (98.0 ± 70.0 mGycm^2^) as compared with that in the Freeze 3D (69.0 ± 56.0 mGycm^2^) and RF groups (74.0 ± 73.0 mGycm^2^, both p < 0.05). Moreover, perioperative complications such as phrenic nerve injury and vascular injuries were not significantly different among the three groups.

**Table 1:** Baseline and perioperative characteristics of AF patients assigned to different groups according to the applied AF ablation strategy.

	Freeze	Freeze 3D	RF	p
Age (years)	59.4 ± 11.3	60.2 ± 10.2	57.7 ± 10.0	0.530
Male, n (%)	38 (54.3)	43 (61.4)	41(58.6)	0.440
Hypertension, n (%)	44 (62.9)	41 (58.6)	35 (54.3)	0.147
Diabetes, n (%)	10 (14.3)	12 (17.1)	15 (21.4)	0.637
MI, n (%)	2 (2.8)	1 (1.4)	0 (0)	0.202
HF, n (%)	5 (7.1)	5 (7.1)	5 (7.1)	1.000
Current smoker, n (%)	8 (11.4)	13 (18.6)	16 (22.9)	0.107
SBP (mmHg)	130.2 ± 15.2	134.8 ± 18.7	133.7 ± 20.0	0.292
DBP (mmHg)	82.0 ± 12.1	82.4 ± 11.4	83.4 ± 14.6	0.769
FBG (mmol/L)	5.1 ± 1.6	5.0 ± 0.8	5.1 ± 1.2	0.990
HbA1c (%)	6.4 ± 1.7	6.0 ± 1.0	6.2 ± 1.3	0.597
Serum potassium (mmol/L)	4.0 ± 0.4	3.9 ± 0.3	4.0 ± 0.4	0.306
Serum sodium (mmol/L)	142.1 ± 1.8	142.4 ± 2.1	141.8 ± 2.2	0.330
SCr (umol/L)	71.8 ± 14.6	70.7 ± 17.6	69.1 ± 15.2	0.652
BUN (mmol/L)	4.9 ± 1.2	5.3 ± 1.7	5.2 ± 1.4	0.345
Hcy (umol/L)	17.1 ± 5.3	20.3 ± 12.7	16.2 ± 9.7	0.183
Cystatin C (umol/L)	1.1 ± 0.2	1.1 ± 0.2	1.3 ± 1.6	0.786
Surgical X-ray (mGycm^2^)	98.0 ± 70.0	69.0 ± 56.0	74.0 ± 73.0	0.021
Phrenic nerve injury, n (%)	1 (1.4)	1 (1.4)	0 (0)	0.604
Vascular injuries, n (%)	1 (1.4)	0 (0)	2 (2.9)	0.363

AF, atrial fibrillation; MI, myocardial infarction; HF, heart failure; SBP, systolic blood pressure; DBP, diastolic blood pressure; FBG, fasting blood glucose; HbA1c, glycosylated hemoglobin; SCr, serum creatinine; BUN, blood urea nitrogen; Hcy, homocysteine.

### Effects of different ablation strategies on long-term LA function

**Figure 1:**
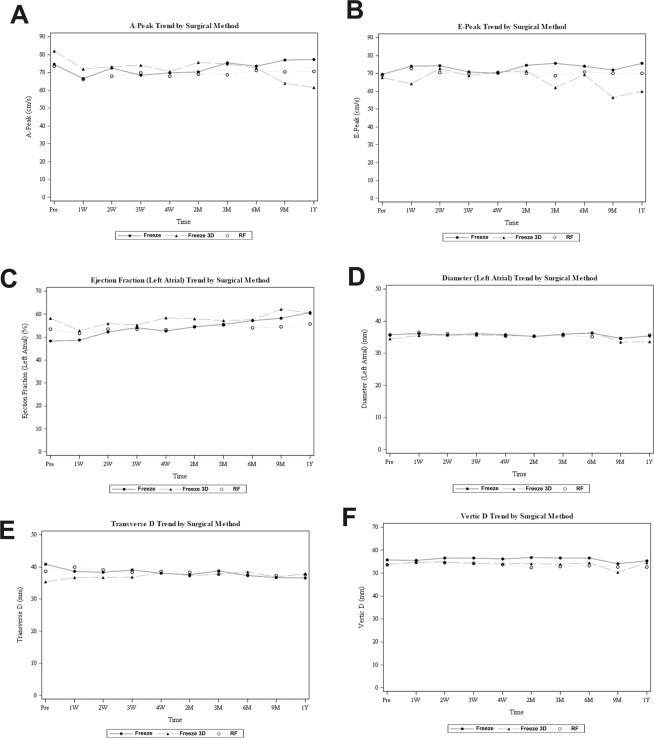
Effects of different ablation strategies on long-term LA function as evaluated by conventional echocardiography following the ablation procedures; (**A**) changes in peak A-wave velocity; (**B**) changes in peak E-wave velocity; (**C**) changes in LAEF; (**D**) changes in LA anteroposterior dimension; (**E**) changes in LA transverse dimension; (**F**) changes in LA vertical dimension.

The temporal changes in peak A-wave velocity did not differ significantly among the three groups (p = 0.22, Table 2 and Fig. 1A). Therefore, we observed trends in temporal changes in peak A-wave velocity with a combination of the three ablation groups. A significant decrease in the peak A-wave velocity was observed at day 7 after ablation in the patients in different ablation groups (p < 0.001 compared with the baseline), followed by gradual recovery beginning from 1–3 months after ablation, and to a similar level as compared with the baseline at 9–12 months after ablation (P = 0.061 and 0.961, respectively, for peak A-wave velocity at 9 and 12 months as compared with the baseline). Moreover, the temporal changes in peak E-wave velocity did not differ significantly among the three groups (p = 0.237, Table 2 and Fig. 1B), while the levels of peak E-wave velocity with a combination of the three ablation groups were stable within 1 year of follow-up (p > 0.05 for all follow-up time points compared to baseline, Table 2). For LAEF, an insignificant increasing trend was observed in patients allocated to the Freeze group (baseline: 48.3 ± 17.5% vs 12 months: 60.7 ± 7.5%, p = 0.136). However, the LAEF remained stable in patients allocated to the Freeze 3D and RF groups, and the temporal changes in LAEF did not differ significantly among the three groups (Table 2 and Fig. 1C). The parameters of LA dimensions (including the anteroposterior, transverse, and vertical dimensions) were stable for patients in all three groups, and the temporal changes in the LA dimensions did not differ significantly (all p > 0.05, Table 2 and Fig. 1D–F).

**Table 2:** Changes of parameters of LA function as evaluated by conventional echocardiography following the ablation procedures.

Variables	Groups	Baseline	1 week	2 weeks	3 weeks	4 weeks	2 months	3 months	6 months	9 months	12 months	p for groups
Peak A-wave velocity (cm/s)	Freeze	74.62 ± 20.33	66.54 ± 19.10	72.51 ± 22.30	68.37 ± 20.34	69.76 ± 22.65	70.23 ± 23.53	75.23 ± 20.94	73.55 ± 18.46	77.00 ± 17.20	77.33 ± 17.64	0.220
	Freeze 3D	82.12 ± 21.48	71.98 ± 22.66	73.17 ± 25.54	74.13 ± 19.54	70.43 ± 19.87	75.57 ± 19.30	74.85 ± 21.16	72.50 ± 22.56	64.00 ± 16.46	61.40 ± 10.26	
	RF	73.37 ± 19.97	65.99 ± 17.31	67.84 ± 17.62	69.76 ± 21.14	67.96 ± 19.34	68.88 ± 20.48	68.67 ± 20.04	71.02 ± 19.16	70.20 ± 18.46	70.58 ± 15.70	
	p for time	—	<0.001	<0.001	<0.001	<0.001	<0.001	0.006	0.002	0.061	0.961	
Peak E-wave velocity (cm/s)	Freeze	69.50 ± 21.80	74.07 ± 22.71	74.26 ± 21.81	70.83 ± 18.28	69.92 ± 18.60	74.50 ± 23.27	75.51 ± 20.37	73.95 ± 19.32	71.95 ± 26.08	75.61 ± 17.31	0.237
	Freeze 3D	67.72 ± 19.82	64.14 ± 18.12	72.69 ± 21.93	68.64 ± 18.49	70.88 ± 25.10	71.43 ± 22.79	62.05 ± 15.22	69.16 ± 22.17	56.57 ± 8.52	60.00 ± 7.52	
	RF	68.86 ± 18.98	72.61 ± 19.21	70.59 ± 21.46	69.78 ± 18.55	70.62 ± 21.79	69.90 ± 20.90	68.81 ± 19.69	70.93 ± 17.43	69.95 ± 18.23	70.11 ± 17.20	
	p for time	—	0.518	0.222	0.986	0.887	0.211	0.928	0.501	0.999	0.164	
LAEF (%)	Freeze	48.26 ± 17.50	48.70 ± 13.70	52.22 ± 13.68	54.06 ± 12.20	52.52 ± 15.21	54.41 ± 14.76	55.54 ± 15.63	57.19 ± 12.98	58.28 ± 8.16	60.69 ± 7.52	0.131
	Freeze 3D	58.21 ± 14.78	52.75 ± 11.73	56.00 ± 12.05	55.28 ± 12.64	58.48 ± 11.21	57.95 ± 12.33	57.18 ± 11.25	57.47 ± 8.44	62.14 ± 8.67	60.50 ± 8.54	
	RF	53.42 ± 13.90	51.51 ± 11.76	53.51 ± 11.62	53.49 ± 10.51	53.24 ± 12.91	54.43 ± 12.20	55.34 ± 11.05	54.06 ± 13.27	54.39 ± 10.22	55.62 ± 13.25	
	p for time	—	0.124	1.000	0.99	0.898	0.567	0.162	0.178	0.304	0.112	
LA anteroposterior dimension (mm)	Freeze	35.80 ± 4.42	36.11 ± 3.46	35.65 ± 3.53	36.04 ± 3.79	35.76 ± 3.46	35.28 ± 3.28	35.91 ± 3.11	36.27 ± 3.49	34.60 ± 2.19	35.35 ± 5.11	0.608
	Freeze 3D	34.52 ± 4.25	35.53 ± 4.48	35.66 ± 4.16	35.69 ± 4.47	35.49 ± 4.19	35.41 ± 4.47	35.79 ± 4.76	36.58 ± 4.87	33.50 ± 3.89	33.60 ± 3.29	
	RF	35.67 ± 5.05	36.58 ± 4.73	36.06 ± 4.17	35.81 ± 4.42	35.39 ± 4.17	35.31 ± 4.29	35.44 ± 4.57	35.19 ± 3.95	34.55 ± 3.49	35.58 ± 6.92	
	p for time	—	0.620	0.870	0.283	0.940	0.997	1.000	0.973	1.000	1.000	
LA transverse dimension (mm)	Freeze	40.87 ± 6.93	38.67 ± 4.86	38.25 ± 6.48	39.00 ± 4.88	37.98 ± 5.03	37.57 ± 3.81	38.73 ± 3.79	37.26 ± 4.32	36.70 ± 2.79	36.56 ± 3.48	0.209
	Freeze 3D	35.31 ± 3.71	36.62 ± 3.74	36.71 ± 3.30	36.77 ± 4.46	38.13 ± 6.98	37.24 ± 4.03	37.65 ± 5.16	38.53 ± 4.29	36.86 ± 2.61	37.80 ± 2.39	
	RF	38.66 ± 7.80	39.88 ± 8.09	39.11 ± 4.93	38.35 ± 4.56	38.65 ± 5.26	38.27 ± 4.61	37.81 ± 5.18	37.43 ± 3.48	37.24 ± 4.09	37.57 ± 6.12	
	p for time	—	1.000	0.984	0.866	1.000	0.444	0.834	0.233	0.325	0.505	
LA vertical dimension (mm)	Freeze	55.63 ± 7.65	55.57 ± 6.20	56.49 ± 6.44	56.48 ± 6.87	56.18 ± 6.31	56.68 ± 6.25	56.48 ± 6.20	56.44 ± 5.32	54.00 ± 3.24	55.39 ± 5.01	0.369
	Freeze 3D	53.63 ± 5.62	54.67 ± 6.12	54.80 ± 5.99	54.36 ± 6.75	54.06 ± 7.56	53.95 ± 6.25	53.85 ± 6.40	54.47 ± 5.72	50.43 ± 5.06	54.40 ± 7.50	
	RF	53.72 ± 6.44	54.82 ± 5.88	54.43 ± 5.13	54.30 ± 5.93	53.71 ± 5.91	52.30 ± 6.25	52.87 ± 5.79	53.31 ± 6.17	52.50 ± 5.50	52.61 ± 6.49	
	p for time	—	0.411	0.318	0.455	1.000	0.999	0.975	1.000	0.986	1.000	

p for groups indicates the p values for the comparisons of the temporal changes of related parameters among the three ablation groups.

p for time indicates the p values for the comparisons for the related parameters to their baseline values at individual follow-up time point with the combination of the three ablation groups.

**Figure 2:**
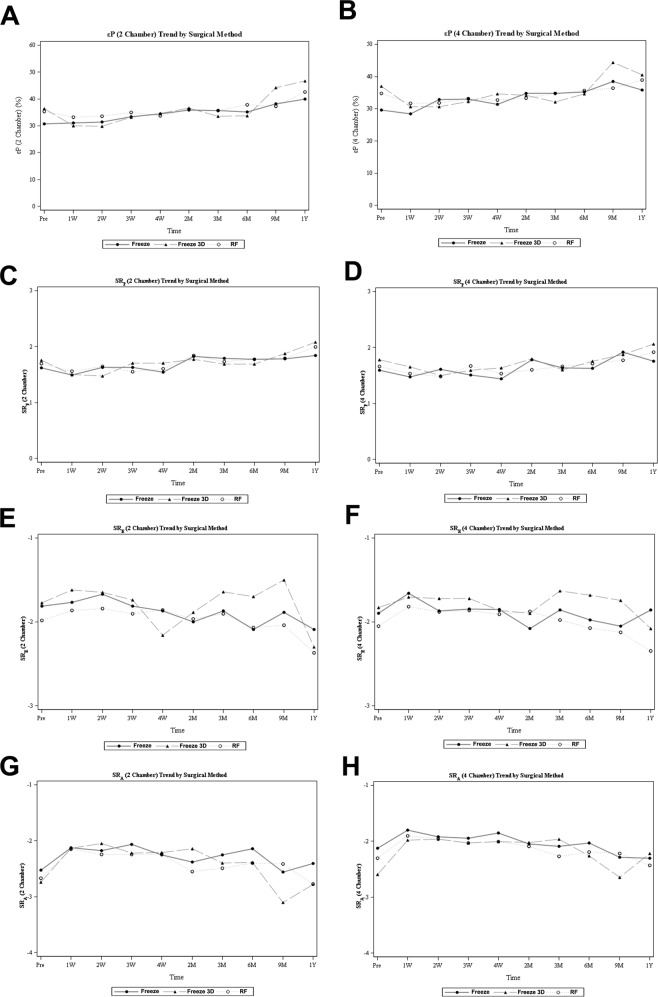
Effects of different ablation strategies on long-term LA function as evaluated by speckle tracking analyses following the ablation procedures; (**A,B**) changes in LA peak systolic strain in two- and four-chamber views; (**C,D**) changes in LA peak systolic strain rate in two- and four-chamber views; (**E,F**) changes in LA peak early diastolic strain rate in two- and four-chamber views; (**G,H**) changes in LA peak late diastolic strain rate in two- and four-chamber views.

The temporal changes in the εP, SR_P_, SR_E_ and SR_A_ at both the two-chamber and four-chamber views did not differ significantly among the three groups within 1 year after PAF ablation (all p > 0.05). Therefore, we observed trends in temporal changes of the above parameters with a combination of the three ablation groups. We found that the εP, SR_P_, SR_E_ and SR_A_ at both the two-chamber and four-chamber views were significantly decreased at day 7 after the ablation procedures for included patients with the combination of the three ablation groups (p < 0.05 for all parameters at 1 week compared with the baseline, Table 3 and Fig. 2A–H). These parameters then remained stable before recovering gradually within 4 weeks to 3 months after ablation in the strain analyses performed at both the two-chamber and four-chamber views (Table 3 and Fig. 2A–H). Full recoveries of these parameters to levels similar to baseline were observed 9 months after ablation (p > 0.05 for all parameter at 9 months after ablation as compared with their individual levels at baseline, Table 3 and Fig. 2A–H).

**Table 3:** Changes of parameters of LA function as evaluated by speckle tracking analyses following the ablation procedures.

Variables	Groups	Baseline	1 week	2 weeks	3 weeks	4 weeks	2 months	3 months	6 months	9 months	12 months	p for groups
LA peak systolic strain (2 Chamber)	Freeze	30.67 ± 13.34	31.02 ± 9.76	31.41 ± 10.27	33.32 ± 11.95	34.36 ± 11.80	35.79 ± 15.54	35.74 ± 12.14	35.17 ± 12.36	38.20 ± 11.56	39.88 ± 9.74	0.471
	Freeze 3D	36.33 ± 13.44	30.04 ± 10.54	29.92 ± 8.25	33.21 ± 11.07	34.62 ± 10.87	36.65 ± 14.30	33.63 ± 11.00	33.68 ± 9.81	44.14 ± 16.01	46.60 ± 15.92	
	RF	35.40 ± 11.80	33.14 ± 10.18	33.53 ± 9.45	34.93 ± 11.99	33.72 ± 9.88	36.21 ± 10.86	35.72 ± 10.47	37.83 ± 11.66	37.24 ± 8.57	42.58 ± 16.32	
	p for time	—	0.005	0.021	0.990	0.998	0.797	1.000	1.000	0.603	0.005	
LA peak systolic strain (4 Chamber)	Freeze	29.57 ± 10.87	28.48 ± 10.17	32.85 ± 9.25	33.00 ± 11.44	31.40 ± 9.81	34.77 ± 16.41	34.70 ± 11.16	35.28 ± 12.49	38.47 ± 9.34	35.75 ± 7.30	0.548
	Freeze 3D	36.93 ± 13.36	30.60 ± 11.91	30.64 ± 10.85	32.32 ± 11.24	34.67 ± 12.96	34.14 ± 12.52	32.17 ± 12.22	34.58 ± 9.36	44.43 ± 10.75	40.60 ± 7.57	
	RF	34.82 ± 13.15	31.72 ± 10.45	31.82 ± 8.32	33.13 ± 11.77	32.67 ± 9.65	33.35 ± 10.80	34.76 ± 9.21	35.60 ± 10.54	36.39 ± 9.65	38.92 ± 17.03	
	p for time	—	<0.001	0.049	0.479	0.514	0.969	0.999	1.000	0.608	0.574	
LA peak diastolic strain (2 Chamber)	Freeze	1.62 ± 0.74	1.49 ± 0.53	1.63 ± 0.58	1.63 ± 0.60	1.54 ± 0.47	1.82 ± 0.65	1.79 ± 0.64	1.78 ± 0.59	1.78 ± 0.55	1.84 ± 0.43	0.567
	Freeze 3D	1.76 ± 0.78	1.50 ± 0.51	1.48 ± 0.41	1.71 ± 0.55	1.71 ± 0.49	1.77 ± 0.71	1.68 ± 0.90	1.68 ± 0.69	1.87 ± 0.48	2.08 ± 1.23	
	RF	1.70 ± 0.56	1.56 ± 0.54	1.65 ± 0.66	1.55 ± 0.68	1.60 ± 0.46	1.84 ± 0.70	1.73 ± 0.59	1.77 ± 0.56	1.79 ± 0.44	1.99 ± 0.70	
	p for time	—	0.040	0.534	0.215	0.389	0.999	1.000	0.992	0.883	1.000	
LA peak diastolic strain (4 Chamber)	Freeze	1.59 ± 0.84	1.48 ± 0.47	1.61 ± 0.46	1.51 ± 0.52	1.44 ± 0.43	1.78 ± 1.02	1.64 ± 0.53	1.63 ± 0.41	1.92 ± 0.54	1.76 ± 0.43	0.183
	Freeze 3D	1.78 ± 0.64	1.66 ± 0.65	1.50 ± 0.54	1.59 ± 0.54	1.63 ± 0.80	1.80 ± 0.99	1.60 ± 0.67	1.75 ± 0.68	1.87 ± 0.55	2.06 ± 0.78	
	RF	1.66 ± 0.61	1.53 ± 0.61	1.49 ± 0.50	1.67 ± 0.62	1.53 ± 0.46	1.61 ± 0.49	1.66 ± 0.53	1.72 ± 0.55	1.77 ± 0.41	1.92 ± 0.71	
	p for time	—	0.038	0.466	1.000	0.97	1.000	0.998	1.000	0.987	0.928	
LA peak early diastolic strain rate (2 Chamber)	Freeze	−1.81 ± 0.74	−1.77 ± 0.54	−1.67 ± 0.74	−1.81 ± 0.84	−1.87 ± 0.98	−2.00 ± 1.05	−1.87 ± 0.72	−2.09 ± 1.26	−1.89 ± 0.65	−2.09 ± 0.64	0.366
	Freeze 3D	−1.77 ± 1.00	−1.62 ± 0.66	−1.65 ± 0.71	−1.74 ± 0.74	−2.16 ± 2.95	−1.88 ± 0.86	−1.64 ± 0.66	−1.70 ± 0.74	−1.50 ± 2.08	−2.30 ± 1.12	
	RF	−1.98 ± 0.78	−1.86 ± 0.69	−1.84 ± 0.68	−1.90 ± 0.86	−1.86 ± 0.72	−1.97 ± 0.85	−1.91 ± 0.96	−2.07 ± 1.00	−2.04 ± 0.77	−2.37 ± 1.20	
	p for time	—	0.041	0.228	0.924	1.000	1.000	0.911	1.000	0.984	0.587	
LA peak early diastolic strain rate (4 Chamber)	Freeze	−1.90 ± 0.75	−1.66 ± 0.48	−1.87 ± 0.63	−1.85 ± 0.75	−1.85 ± 0.70	−2.08 ± 1.15	−1.86 ± 0.71	−1.97 ± 0.83	−2.05 ± 0.60	−1.86 ± 0.62	0.092
	Freeze 3D	−1.83 ± 0.69	−1.71 ± 0.84	−1.72 ± 0.73	−1.72 ± 0.70	−1.87 ± 0.76	−1.90 ± 0.83	−1.63 ± 0.66	−1.68 ± 0.76	−1.74 ± 1.35	−2.08 ± 0.48	
	RF	−2.05 ± 0.90	−1.82 ± 0.87	−1.88 ± 0.78	−1.86 ± 0.73	−1.91 ± 0.73	−1.87 ± 0.81	−1.98 ± 0.78	−2.07 ± 0.81	−2.12 ± 0.94	−2.34 ± 1.14	
	p for time	—	< 0.001	0.158	0.043	0.272	0.784	0.101	0.955	0.831	1.000	
LA peak late diastolic strain rate (2 Chamber)	Freeze	−2.53 ± 1.29	−2.13 ± 0.89	−2.18 ± 0.84	−2.07 ± 1.10	−2.25 ± 0.92	−2.38 ± 1.06	−2.25 ± 0.89	−2.14 ± 0.76	−2.56 ± 1.08	−2.41 ± 0.65	0.380
	Freeze 3D	−2.74 ± 1.00	−2.13 ± 0.65	−2.05 ± 0.61	−2.22 ± 0.84	−2.21 ± 0.85	−2.14 ± 1.86	−2.40 ± 0.62	−2.39 ± 0.80	−3.10 ± 1.39	−2.78 ± 0.88	
	RF	−2.67 ± 1.02	−2.15 ± 0.71	−2.24 ± 0.77	−2.24 ± 0.87	−2.24 ± 0.80	−2.55 ± 1.00	−2.49 ± 0.86	−2.39 ± 0.78	−2.41 ± 0.49	−2.77 ± 1.09	
	p for time		<0.001	<0.001	<0.001	<0.001	0.002	0.006	<0.001	0.153	0.299	
LA peak late diastolic strain rate (4 Chamber)	Freeze	−2.12 ± 0.90	−1.80 ± 0.92	−1.92 ± 0.62	−1.94 ± 0.69	−1.85 ± 0.65	−2.05 ± 1.35	−2.09 ± 1.20	−2.03 ± 0.84	−2.29 ± 0.74	−2.30 ± 0.52	0.569
	Freeze 3D	−2.59 ± 1.10	−1.98 ± 0.79	−1.96 ± 0.79	−2.03 ± 0.78	−2.01 ± 0.63	−2.02 ± 0.84	−1.96 ± 1.14	−2.26 ± 0.56	−2.64 ± 0.30	−2.22 ± 0.31	
	RF	−2.30 ± 0.89	−1.91 ± 0.74	−1.96 ± 0.66	−2.03 ± 0.76	−2.00 ± 0.67	−2.09 ± 0.99	−2.27 ± 0.80	−2.20 ± 0.71	−2.22 ± 0.76	−2.43 ± 1.16	
	p for time		0.029	0.087	0.171	0.130	0.432	0.030	0.909	1.000	1.000	

p for groups indicates the p values for the comparisons of the temporal changes of related parameters among the three ablation groups.

p for time indicates the p values for the comparisons for the related parameters to their baseline values at individual follow-up time point with the combination of the three ablation groups.

### Effects of different ablation strategies on recurrences of ATA

Recurrences of ATA in patients from each group within 12 months after PAF ablation are shown in Table 4. Briefly, the recurrence of AF seemed to be lower in patients received 3D mapping-guided cryoablation (9.5%) as compared with that in those who received cryoablation (16.0%) or RFCA (20.4%). However, the incidence of ATA did not differ significantly among the three groups (p = 0.17).

**Table 4:** Recurrence of ATA within 12 months after AF ablation in different groups.

	Freeze	Freeze 3D	RF	p
PAC	1 (1.5)	0 (0)	1 (1.3)	0.604
AF	11 (16.0)	6 (9.5)	14 (20.4)	0.157
AT	2 (2.5)	2 (2.2)	0 (0.0)	0.361
A Flutter	0 (0)	1 (1.1)	0 (0)	0.366

AF, atrial fibrillation; PAC, premature atrial contraction; AT, atrial tachycardia.

### Effects of different ablation strategies on perioperative myocardial injury and systematic inflammation

**Figure 3:**
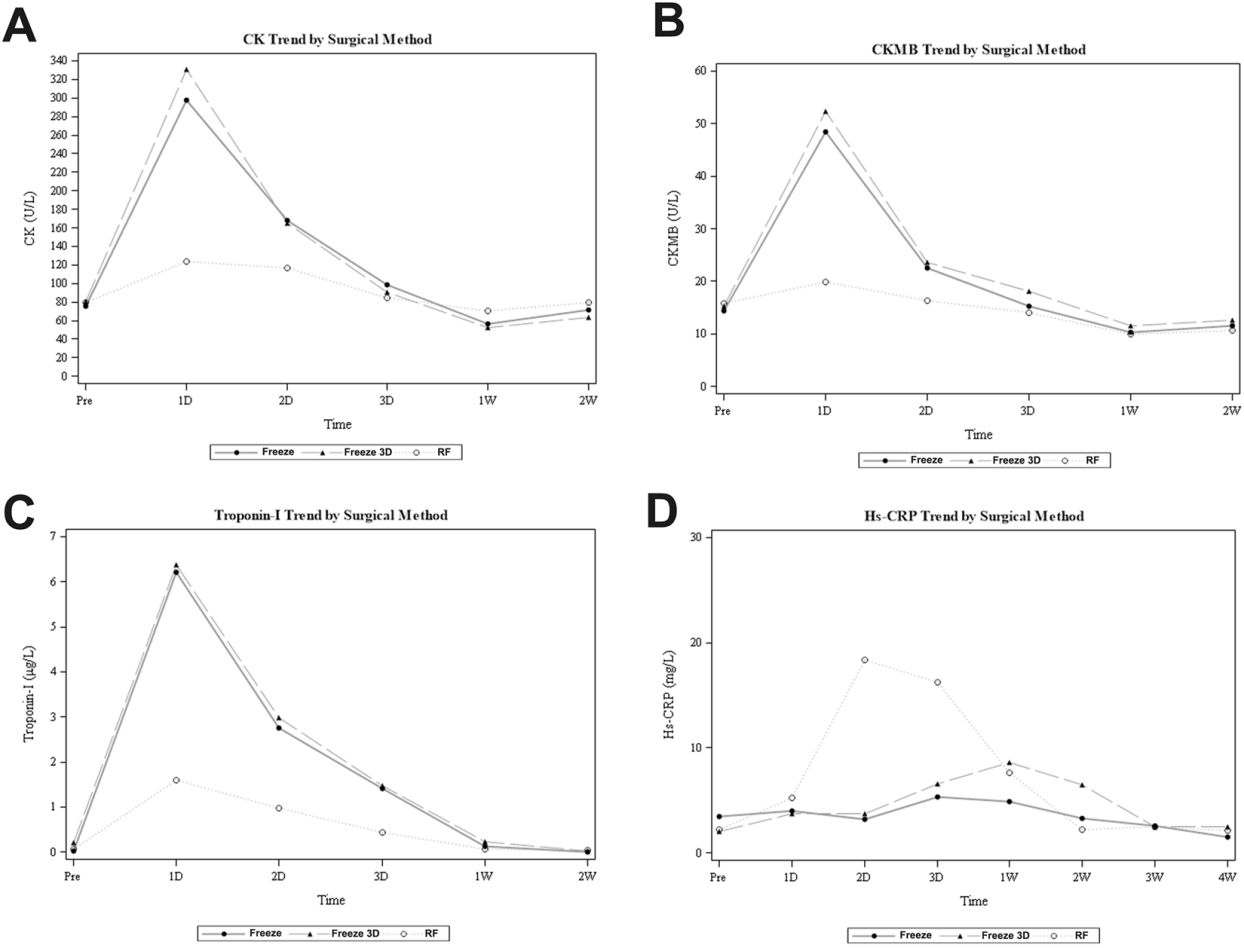
Effects of different ablation strategies on perioperative biomarkers of myocardial injury and systematic inflammation; (**A**) perioperative changes in CK; (**B**) perioperative changes in CK-MB; (**C**) perioperative changes in troponin I; (**D**) perioperative changes in hs-CRP.

The temporal changes in circulating indexes of myocardial injury (CK, CKMB, and troponin I) and systematic inflammation (hs-CRP) within 4 weeks after PAF ablation are shown in Fig. 3. These results showed that for patients allocated to the Freeze and Freeze 3D groups, all three biomarkers of myocardial injury dramatically increased to peak levels of about 4–6 times baseline levels by 1 day after ablation and were then maintained above the baseline levels for up to 1–2 weeks after ablation (Fig. 3A–C). For patients allocated to the RF group, the increments of the three biomarkers of myocardial injury were significantly less as compared to those for patients allocated to the Freeze and Freeze 3D groups (all p < 0.001). Interestingly, we found that the temporal increase in hs-CRP was significantly greater in the RF group compared with the Freeze and Freeze 3D groups (both p < 0.001), with a peak of almost 4 times the baseline level at 2 days after ablation, and the increase was maintained for 2 weeks (Fig. 3D). The temporal increments in hs-CRP in the Freeze and Freeze 3D groups were not significant as compared with the baseline levels (both p > 0.05).

## Discussion

In this study, we found that for PAF patients undergoing catheter ablation, a transient decline in LA function occurred within 7 days to 3 months after ablation, which is consistent with previous notions that LA function is stunned after the restoration of sinus rhythm in AF. Moreover, we found that the temporary changes in LA function as evaluated by conventional and sparkle tracking echocardiography were not significantly different among patients who received ablation via different strategies, including conventional RFCA, novel cryoablation, as well as 3D mapping-guided cryoablation complemented by RFCA for additional points. Interestingly, we also found that although cryoablation was associated with more complete myocardial damage as compared with RFCA, systematic inflammation was not significantly induced. Moreover, pilot results showed that the three ablation strategies did not show significant differences regarding the recurrence of ATA within 1 year or the incidence of severe complications during the perioperative period. Based on the above findings, we conclude that PAF ablation with RFCA, cryoablation, or 3D mapping-guided cryoablation did not differentially influence long-term atrial function after AF ablation. The value of 3D mapping-guided cryoablation for PAF remains uncertain at the current stage.

One of the important strengths of our study is the comprehensive evaluation of the temporal changes in LA function following PAF ablation in patients who received ablation using different strategies. Despite the use of the conventional echocardiographic measurements of LA function based on pulsed-wave Doppler techniques, we also applied speckle tracking analyses to dynamically evaluate the movements of the atrial myocardium. This technique has been recommended as a novel measurement method for LA function because it is angle-independent, real-time, and quantitatively reflective of the global and strain systolic and diastolic function of LA^12–14^. We found a trend of transient decreases in peak A-wave velocity, global LA peak systolic strain, peak systolic strain rate, peak early diastolic strain rate, and peak late diastolic strain rate from 7 days to 3 months after ablation followed by gradual recovery of these parameters, indicating the occurrence of LA stunning in these patients during this period. These results confirm our previous findings that for PAF patients who receive RFCA, reduced LA function could be observed within 7 days after restoration of sinus rhythm^6^. Moreover, we expanded these findings by showing that LA stunning, characterized by reduced LA systolic and diastolic function, could be observed within 7 days after cryoablation or cryoablation guided by 3D mapping. These results may reflect the previous notion that LA stunning is a common outcome of sinus restoration rather than a feature of a certain ablation strategy. Interestingly, in view of the fact that LA stunning has been related to the risk of thromboembolism events^5^ and the relapse into AF^15^ after sinus restoration, the results of our study may indicate that the risk of thromboembolism events related to AF recurrence may not differ fundamentally in patients who are treated using the three different treatment strategies. This has been confirmed by the results of the Fire and Ice study^9^ and recent meta-analyses^16–18^. Taken together, these results demonstrate that the temporal changes in LA function after ablation are not significantly affected by the ablation strategies, highlighting the importance of adequate anticoagulation therapy and rhythm monitoring despite the immediate success of AF ablation in these patients.

Another interesting finding of our study is that although cryoablation was associated with more complete myocardial damage as compared with RFCA, systematic inflammation was not significantly induced. This finding is consistent with the results of a previous study in Europe, which also showed that cryoballoon ablation of AF causes more significant myocardial damage but does not induce a significantly greater inflammatory response as compared with RFCA^19^. These findings may be reflective of the unique feature of cryothermal energy-induced histological changes of the myocardium. Changes in myocardial tissues in response to cryoablation, as compared with RFCA, include ice crystal formation and preserved tissue architecture, but more extensive ablation lesions to the myocardium^20^. However, the clinical relevance of more complete myocardial ablation but relieved systematic inflammation caused by cryoablation remains to be understood.

In view of previous reports that considerable patients experience incomplete PVI despite the use of the circular mapping catheter to record the LA-PV potential connections during cryoablation for AF^10,11^, we designed a 3D mapping-guided cryoablation complemented by RFCA for additional points. Although we did not find improvements in atrial function or ATA recurrence during follow-up in patients allocated to the 3D mapping-guided cryoablation group, the incidence of AF recurrence seemed to be lower in patients who received 3D mapping-guided cryoablation (9.5%) as compared with that among those who received cryoablation (16.0%) or RFCA (20.4%). Studies aiming to improve the success of cryoablation for AF by using mapping-guided ablation are rare. In a cohort study from the USA, using of CARTO or NavX mapping system as a guiding strategy to cryoballoon ablation yielded longer fluoroscopy times without an improvement in AF recurrence^21^. However, another pilot study using a high-resolution mapping system during cryoablation for AF significantly improved the detection of incomplete PVI, which is expected to reduce the incidence of unrecognized incomplete PVI-related recurrence of AF^11^. Overall, the optimal strategy for guided cryoablation and its potential value in clinical practice remain uncertain at present, and more clinical trials are warranted.

Our study has limitations that should be noticed when interpreting the results. Firstly, our study was designed to evaluate the effects of different ablation strategies on LA function rather than on clinical outcomes such as AF recurrence. Therefore, large-scale trials are needed to evaluate the influences of different ablation strategies on clinical outcomes such as thromboembolism events and AF recurrence in the future. Secondly, use of 3D mapping-guided cryoablation leads to additional expense. The most cost-effective ablation strategy for PAF patients remains to be determined. Thirdly, the study was a single blind clinical trial, which could lead to additional bias, especially considering it involved a small number of patients. Fourthly, the application of RFCA for patients in the Freeze 3D group may also have confounded the outcome of LA function in this study. Finally, this was a single-center study. The results of our study should be further confirmed by large-scale multi-center studies.

In conclusion, the results of our study showed that LA stunning occurred within 7 days to 3 months after ablation regardless of the ablation strategy applied, and different strategies of AF ablation did not significantly affect the temporal changes in LA function for up to 1 year. Further studies are needed to determine the optimal strategy to guide cryoablation and its potential value in clinical practice.

## Methods

This study was designed to evaluate the potential influence of three different ablation strategies (cryoablation, cryoablation combined with 3D mapping, and RFCA), applied with and without perioperative statins, on the long-term LA function in PAF patients. Therefore, six groups were allocated (three ablation arms with and without statins), and in this paper, we focused on the reporting of the influences of the ablation strategies on LA function. The protocol of this single blind, randomized controlled trial was approved by the Ethics Committee of the Second Hospital of Hebei Medical University, and we hereby confirm that all methods were performed according to the guidelines and regulations approved by the Ethics Committee. Informed consent from the included patient was required. Participation in the study was voluntary and the patient could withdraw their consent at any time. The study protocol was registered at Chinese Clinical Trial Registry (http://www.chictr.org.cn, No. 16007954, firstly registered on 20/02/2016). All the methods were conducted according to the CONSORT statement^22^.

### Inclusion and exclusion criteria

Consecutive patients undergoing catheter ablation for AF in our center between March 1, 2016 and March 1, 2017 were screened for possible participation. Patients were included if they met the following criteria: (1) ECG-confirmed PAF that occurred at least twice within 6 months before study enrollment; (2) occurrence of PAF remained despite application of class I and III antiarrhythmic drugs; and (3) <80 years old and agreed to receive catheter ablation treatment for PAF. Patients with the following clinical statuses were excluded: (1) prior history of receiving catheter ablation for AF; (2) atrial thrombosis; (3) diagnosis of valvular heart disease (moderate and severe valvular stenosis, severe valvular regurgitation); (4) an LA dimension of >50 mm; (5) prior history of prosthetic heart valve replacement; (5) pregnancy; or (6) existing liver and kidney diseases, malignant tumors or hematological system diseases.

### Groups and randomization

Patients were randomized to one of the following groups according to computer generated random sequences: (1) the cryoablation group (Freeze group): AF ablation was achieved via a standardized protocol with the first or second generation cryoballoon; repeated cryoablation was performed for each pulmonary vein until complete PVI was achieved; (2) the cryoablation combined with 3D mapping group (Freeze 3D group): cryoablation was performed a maximum of two times for each pulmonary vein under guidance with a 3D mapping system with the first or the second generation cryoballoon; if PVI was not achieved, RFCA was applied for the additional points until complete PVI was achieved; and (3) the RFCA group (RF group): AF ablation was achieved via a standardized RFCA procedure. The clinical characteristics and routine biochemical indexes of each patient were obtained before the ablation procedures. Only the physicians who performed the ablation procedures were aware of the grouping of the patients.

### Ablation procedures

Reconstructive computed tomography (CT) images of the PV were obtained from all included patients before the performance of the ablation procedure.

For patients allocated to the Freeze group, single-balloon ablation with a cryoballoon either 28 mm or 23 mm in diameter (Arctic Front Advance™ Cardiac CryoAblation Catheter, Medtronic, Minneapolis, MN) was performed according to the results of angiographic images of the PV. Briefly, an inner lumen mapping catheter (Achieve, Medtronic, MN, USA) was placed into each PV ostium. Then, cryoballoons of selected diameters were advanced and placed at each PV ostium. Cryothermal energy was released and the single-time cryoablation was performed for up to 240 s for procedures of the first-generation cryoballoon and up to 180 s for the procedures for the second-generation cryoballoon. The cryoablation could be repeatedly performed until complete isolation was achieved.

For patients allocated to the Freeze 3D group, a circular mapping catheter (Achieve, Medtronic) was used to construct the configuration and build the structures of the left atrium and PV guided by the EnSite NavX 3D mapping system. Then, cryoablation was performed as described in the Freeze group up to two times for each PV. If PVI was not completely achieved, RFCA was applied for the additional points until complete PVI was achieved.

For patients allocated to the RF group, a standardized RFCA procedure was performed with a mapping catheter (Lasso® NAV Eco; Biosense Webster) and a 3D electro-anatomical mapping system (Carto 3; Biosense Webster). The procedures of ablation were performed by a single group of four physicians for all of the included patients according to the randomized group allocation, which was led by a Chief physician (Dr. Ruiqin Xie).

### Follow-up and outcomes

After discharge, the patients were followed up in the clinic at 1, 2, 3, and 4 weeks and 2, 3, 6, 9, and 12 months after the procedure. Patients were followed up for at least 1 year after the ablation for PAF. The primary outcome of the study was the change in LA function as evaluated by real-time echocardiographic examination. Moreover, we evaluated the influence of different ablation strategies on markers of myocardial injury (creatine kinase [CK], creatine kinase-MB [CKMB], and troponin I) and systematic inflammation (high-sensitive C reactive protein, hs-CRP) within 1 month after the procedures. In addition, the influence of different treatment strategies on the recurrence of atrial tachyarrhythmia (ATA) during follow-up was also explored in PAF patients in each group. Recurrent arrhythmia was defined as any ATA lasting for at least 30 s.

### Echocardiographic examination

Echocardiographic examination was performed at baseline, at 1, 2, 3, and 4 weeks, and at 2, 3, 6, 9 and 12 months after PAF ablation using an echocardiographic system (iE33 machines equipped with X3; Philips Medical Systems, Eindhoven, The Netherlands). The echocardiographic examination was performed by one experienced physician who was not aware of the grouping of the patients. Briefly, both the early (E) and late (A) diastolic filling velocities were obtained using pulsed-wave Doppler at the mitral valve leaflet tips. In the apical four-chamber view, the maximal LA volume was measured at the end of the left ventricle (LV) systolic phase just before the opening of the mitral valve, while the minimal LA volume was measured at the end of the LV diastolic phase just after the closure of the mitral valve. Subsequently, the LA emptying fraction (LAEF) was calculated according to the following equation ([maximal LA volume − minimal LA volume]/ maximal LA volume) × 100. The LA maximum anteroposterior diameter was measured in the parasternal long-axis view, while the transverse and vertical diameters of the left atrium were measured in the apical four-chamber view.

### Speckle tracking analyses

Subsequently, we analyzed the longitudinal LA strain using speckle tracking echocardiography (two-dimensional cardiac performance analysis; QLAB software; Philips Medical Systems). Briefly, strain data of the typical 12 LA segments (mid, annular, and superior segments along the lateral, septal, inferior, and anterior LA walls using apical four-chamber and two-chamber images) were averaged to estimate the global LA peak systolic strain (εP) during LV ejection (LA reservoir phase). The peak strain rate (SR_P_), the peak early diastolic strain rate (SRE), and the peak late diastolic strain rate (SRA) were measured during LV ejection, early diastole, and late diastole (occurring after the P wave), which indicate the LA reservoir, LA conduit phase, and LA active contraction phase, respectively.

### Statistical analyses

A sample size of 35 in each of the six arms was found to be sufficient to detect a clinically relevant difference of 2 cm/s of peak A-wave velocity among the groups using a repeated measurements analysis of variance analysis with 95% power and a 5% level of significance. Therefore, a total of 210 patients were needed. Continuous variables are expressed as means ± standard deviations (SDs), and categorical variables are expressed as counts and percentages. Comparisons of continuous variables among the three groups were performed with analysis of variance (ANOVA), and for comparisons of categorical variables, chi-square analyses were applied. Mixed-effects models were constructed when comparing the effect of ablation strategy and time period. The ablation strategy was set to be a fixed effect, and the time period was set to be a random effect as the data were not collected at equal time intervals. For post-hoc analyses, Bonferroni and Dunnet adjustments were used as appropriate. A p value < 0.05 was considered statistically significant. SPSS software (version 13.0, Chicago, IL, USA) was used for statistical analyses.
